# The role of microbial indole metabolites in tumor

**DOI:** 10.1080/19490976.2024.2409209

**Published:** 2024-10-01

**Authors:** Dingjiacheng Jia, Zheng Kuang, Liangjing Wang

**Affiliations:** a Department of Gastroenterology, Second Affiliated Hospital of Zhejiang University School of Medicine, Hangzhou, Zhejiang Province, China; b Department of Biological Sciences, Carnegie Mellon University, Pittsburgh, PA, USA; c Institution of Gastroenterology, Zhejiang University, Hangzhou, Zhejiang Province, China

**Keywords:** Tryptophan, gut microbiota, indole metabolites, ferroptosis, immune checkpoint blockade, tumor chemotherapy, Indole-3-lactic acid, Indole-3-propionic acid

## Abstract

The gut microbiota can produce a variety of microbial-derived metabolites to influence tumor development. Tryptophan, an essential amino acid in the human body, can be converted by microorganisms via the indole pathway to indole metabolites such as Indole-3-Lactic Acid (ILA), Indole-3-Propionic Acid (IPA), Indole Acetic Acid (IAA) and Indole-3-Aldehyde (IAld). Recent studies have shown that indole metabolites play key roles in tumor progression, and they can be used as adjuvant regimens for tumor immunotherapy or chemotherapy. Here, we summarize recent findings on the common microbial indole metabolites and provide a review of the mechanisms of different indole metabolites in the tumor microenvironment. We further discuss the limitations of current indole metabolite research and future possibilities. It is expected that microbial indole metabolites will provide new strategies for clinical therapy.

## 1. Introduction

Cancer continue to threaten people’s health all over the world.^1^ The gut microbiota is a community of microorganisms that reside in the human intestine. Not only are these microbial communities’ important symbiotic inhabitants of our gut, they also seem to play an integral role in the tumor microenvironment.^2,3^ Scientists have used fecal microbiota transplantation (FMT) to manipulate intestinal microecology and tumor development.^4^ And the rapid development of high-throughput bacterial sequencing has led to the culmination of more studies on individual species or strains. So far, some microecology-based clinical trials have begun to move from tumor prevention to adjuvant therapy, such as combined radiotherapy, chemotherapy or immunotherapy.^5–7^

The gut microbiota can metabolize nutrients ingested by the host to produce a range of microbial-derived metabolites.^2^ One of these is tryptophan, an aromatic essential amino acid containing the indole group. Tryptophan is gradually absorbed into the small and large intestines and catabolized by both the host and the microorganisms via three main pathways: the Serotonin pathway, the Kynurenine (Kyn) pathway, and the Indole pathway.^8,9^ Of these, the indole pathway is relatively unique because this pathway consists almost entirely of enzymes generated from microorganisms.

Studies have reported the role of microbial indole metabolites in the development of tumors, and they have also been highlighted as having potential applications in chemotherapy and immunotherapy. Here, we present an overview of indole metabolites that are commonly found in the tumor microenvironment. We introduce the biosynthetic pathways as well as the key enzymes of these metabolites. Then we discuss their roles in the process of tumorigenesis and development, as well as in adjuvant cancer therapy. Finally, we present an outlook on the identification of indole metabolites, clinical and translational applications, mechanistic exploration of indole metabolites, and future research trends.

## 2. The microbial indole pathway in tryptophan metabolism

**Figure 1.**
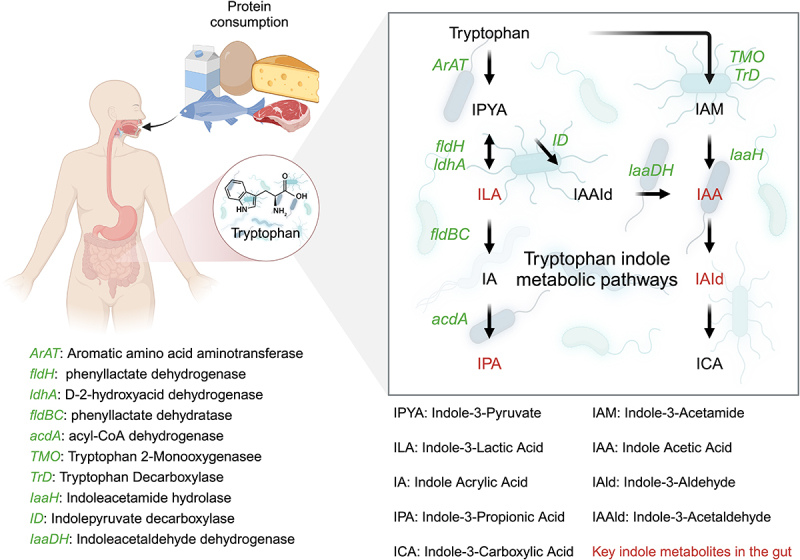
The microbial indole pathway in tryptophan metabolism. Tryptophan-rich foods ingested by the host are converted to indole metabolites by the gut microbiota in the intestine. The indole metabolism pathway has three main components whereby tryptophan is catabolized into key indole metabolites such as ILA, IPA, IAA, and IAld.

A variety of microorganisms have been reported to be able to metabolize tryptophan and produce indole metabolites, particularly members of the *Bacteroides*, *Parabacteroides*, *Bifidobacterium*, *Clostridium*, *Peptostreptococcus*, and *Lactobacillus*.^10–13^ There are differences in the metabolic enzymes contained in different bacteria that contribute to the diversity of indole metabolites. Overall, three main branches of the tryptophan indole pathway are known (Figure 1).

First, tryptophan can be metabolized by aromatic amino acid aminotransferase (*ArAT*) to Indole-3-Pyruvate (IPYA), which is then metabolized by different enzymes in two pathways. Some microorganisms can metabolize IPYA to produce Indole-3-Lactic Acid (ILA), which is further metabolized to Indole Acrylic Acid (IA) and Indole-3-Propionic Acid (IPA). Other bacterial enzymes can metabolize IPYA to Indole-3-Acetaldehyde (IAAld), which is then metabolized to Indole Acetic Acid (IAA), Indole-3-Aldehyde (IAld), and Indole-3-Carboxylic Acid (ICA). In addition, tryptophan can also be metabolized by Tryptophan 2-Monooxygenasee (*TMO*) to Indole-3-Acetamide (IAM) and then to IAA. It is worth noting that the same indole metabolite can be generated by different enzymes in different bacteria. For example, *Clostridium sporogenes* can convert tryptophan to ILA by phenyllactate dehydrogenase (*fldH*), while we recently reported that the enzyme for producing ILA by *Lactobacillus johnsonii* is D-2-hydroxyacid dehydrogenase (*ldhA*).^14^ The isoenzyme activity and substrate specificity for producing the same indole metabolite among different bacteria need to be further clarified.

## 3. Microbial indole metabolites in tumors

Microbial indole metabolites can directly modulate the fate of tumor cells or intervene in the immune microenvironment to indirectly influence tumor development. Some of them also serve as potential adjuvant strategies for tumor immunotherapy or chemotherapy.

### 3.1. Indole metabolites that can act directly on tumor cells

#### 3.1.1. ILA: a star anticancer molecule

**Figure 2.**
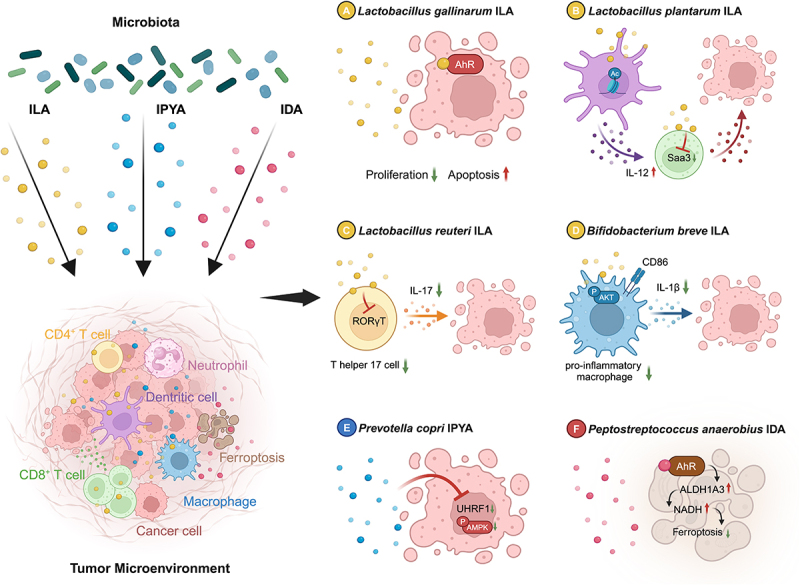
Indole metabolites that can act directly on tumor cells. A. *lactobacillus gallinarum*-derived ILA acts as a ligand for AhR to inhibit tumor cell proliferation and promote apoptosis. B. *lactobacillus plantarum*-derived ILA regulates IL-12 expression in dendritic cells and Saa3 expression in CD8^+^ T cells to kill tumors. C. *lactobacillus reuteri*-derived ILA inhibits IL-17 signaling in Th17 cells to suppress tumors. D. *Bifidobacterium breve*-derived ILA modulates the p-AKT/IL-1b pathway in CD86^+^ pro-inflammatory macrophages to suppress tumors. E. *prevotella copri*-derived IPYA inhibits the UHRF1/p-AMPK axis in tumor cells. F. *peptostreptococcus anaerobius*-derived IDA acts as an AhR ligand to upregulate ALDH1A3/NADH levels and suppress tumor ferroptosis. ILA, Indole-3-lactic Acid; IPYA, Indole-3-Pyruvate; IDA, *trans*-3-indoleacrylic acid.

ILA is the most widely reported indole metabolite with anticancer activity. ILA produced by *Lactobacillus gallinarum* has the ability to directly inhibit colorectal cancer (CRC) cell proliferation and promote apoptosis *in vitro*^15^ (Figure 2A). This mechanism is mediated through the Aryl hydrocarbon receptor (AhR) of tumor cells, and AhR inhibitors counteracts the ability of ILA to inhibit CRC growth *in vivo* and *in vitro*. Various indole metabolites have been shown to be the natural ligands for AhR and can promote its nuclear translocation.^16^

In addition, ILA can also act on a variety of immune cells in the tumor microenvironment, including macrophages, dendritic cells and T cells. *Lactobacillus plantarum* L168-derived ILA can promote the production of IL-12 to activate CD8^+^ T cells by affecting histone modification and chromatin accessibility in dendritic cells, and also promote the release of effectors by inhibiting the key regulatory gene for cholesterol metabolism in CD8^+^ T cells, *Saa3*, thus ultimately combat CRC^17^ (Figure 2B). ILA produced by *Lactobacillus reuteri*, on the other hand, reduces T helper 17 (Th17) cells by inhibiting the transcriptional activity of RORγt, which in turn inhibits CRC^18^ (Figure 2C).

Interestingly, ILA appears to have selective effects on the AhR of different cell types. The authors found that the inhibition of ILA did not require the involvement of the AhR in Th17 cells, but activated the AhR of intestinal epithelial cells to maintain barrier integrity,^18^ and a recent article reported the modulation of macrophage AhR by ILA^19^ (Figure 2D). ILA produced by *Bifidobacterium breve* could reduce the proportion of CD86^+^ pro-inflammatory macrophages through the AhR/p-AKT/IL-1b pathway, which in turn regulated macrophage differentiation and maturation. The authors also found that this effect on macrophage phosphorylation was relatively cell specific, as ILA could not induce AKT phosphorylation in CRC cells.

The broad modulatory effects of ILA on the host immune system are not limited to the tumor microenvironment. ILA production by *Lactobacillus reuteri* was reported to induce intestinal CD4^+^ CD8αα^+^ T cells in germ-free mice.^20^ Furthermore, during the development of the immune system of infants in the first months of life, *Bifidobacterium* species can produce ILA and dose-dependently modulate the immune response of human CD4^+^ T cells and monocytes.^21^ Henrick BM et al. also found that in breast-fed infants, *Bifidobacterium infantis*-derived ILA upregulates the immunoregulatory galectin-1 in Th2 and Th17 cells during polarization.^22^

To summarize, ILA can inhibit tumorigenesis and progression in a direct or indirect manner. Although the current findings are limited to CRC, the broad impact of ILA on the immune microenvironment can also be attempted in other solid tumors. A next question that needs to be addressed is how to increase the level of ILA in the host. Wang L et al. found that the black rice diet rich in dietary fiber and anthocyanins upregulated the abundance of probiotics and increased ILA levels to block colorectal cancer progression.^23^ Supplementation with dietary fiber such as β-glucan has also been found to significantly increase ILA levels.^24^ Intervening in the production of indole metabolites through daily dietary combinations might be a good option in the future.

#### 3.1.2. IPYA: a double-edged sword in tumors?

Indole metabolites are not functionally consistent in tumors. As an intermediate of the indole pathway, IPYA has opposite effects in different types of tumors. *Prevotella copri* has been found to substantially consume host tryptophan, leading to a decrease in IPYA accumulation in the tumor microenvironment^25^ (Figure 2E). Mechanistically, IPYA can directly inhibit the transcription of UHRF1, thereby suppressing the growth of breast and cervical cancer cells in an AMPK signaling pathway-mediated manner. However, a recent study reported that IPYA promotes MYC-driven hepatocellular carcinoma cell growth *in vivo* and *in vitro*.^26^ Another study also found that IPYA acts as a ligand for AhR to enhance glioblastoma migration and inhibit the proliferation of CD8^+^ T cells.^27^ It is noteworthy that in both studies where IPYA was reported to be pro-oncogenic, IPYA was produced by host cells metabolizing tryptophan via interleukin 4-induced 1 (IL4I1), and was not of microbial source. The opposed roles exhibited by different sources of IPYA in different tumor types deserve further experimental clarification and mechanistic resolution.

#### 3.1.3. Indole metabolites related to ferroptosis

Ferroptosis is an iron-dependent, novel mode of programmed cell death, distinct from apoptosis and autophagy, characterized by the accumulation of peroxidized phospholipids.^28^ Tumor cells can escape ferroptosis by limiting free iron and inhibiting peroxide levels.^29,30^ The indole metabolites are known to inhibit ferroptosis. Indole-3-carbinol can act as a ferroptosis inhibitor by scavenging lipid peroxyl radicals.^31^ IPYA has also been reported to inhibit ferroptosis by directly scavenging free radicals and promoting the expression of antioxidant genes.^32^ However, a recent study found that *trans*-3-indoleacrylic acid (IDA) produced by *Peptostreptococcus anaerobius* could inhibit ferroptosis in tumor cells independently of reducing lipid peroxidation (Figure 2F).^33^ IDA acts as a ligand for AhR and upregulates aldehyde dehydrogenase 1 family member A3 (ALDH1A3) expression, which in turn helps ferroptosis-suppressor protein 1 produce reduced coenzyme Q10 to promote CRC progression. As a new target for tumor therapy, more indole metabolites with the ability to regulate ferroptosis need to be further explored.

### 3.2. Modulators of tumor immunotherapy and chemotherapy

#### 3.2.1. IPA: a manipulator of epigenetic modifications

**Figure 3.**
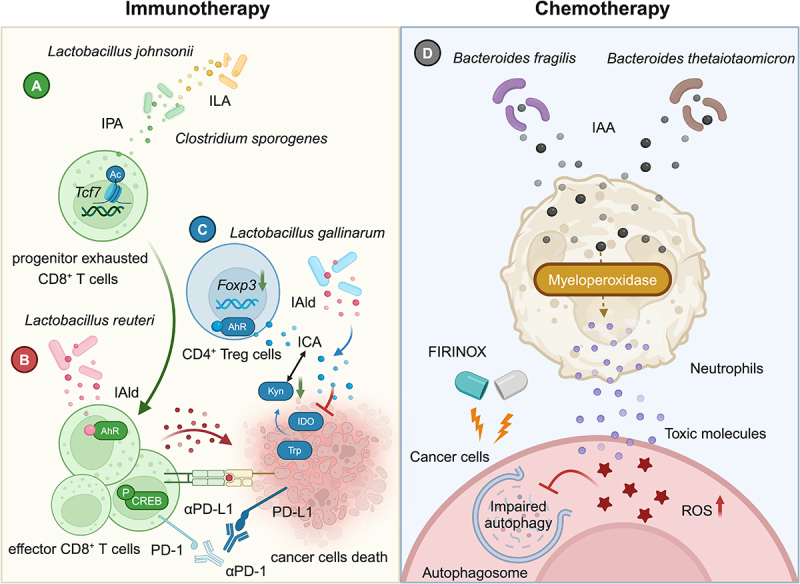
Modulators of tumor immunotherapy and chemotherapy. A. ILA produced by *lactobacillus johnsonii* is metabolized by *Clostridium sporogenes* to IPA, which sensitizes αPD-1 cancer immunotherapy by increasing TCF-1^+^ progenitor exhausted CD8^+^ T cells. B. *lactobacillus reuteri*-derived IAld acts as an AhR ligand dependent on p-creb to sensitize αPD-1 tumor immunotherapy. C. *lactobacillus gallinarum*-derived IAld is metabolized to ICA, which inhibits treg cells and sensitizes αPD-1 cancer immunotherapy by inhibiting Kyn and competing for AhR. D. *bacteroides fragilis* and *bacteroides thetaiotaomicron* produced IAA is metabolized by neutrophils to toxic molecules, modulating the level of ROS and autophagy to sensitize FIRINOX cancer chemotherapy efficacy. ILA, indole-3-lactic Acid; IPA, indole-3-propionic Acid; IAld, indole-3-Aldehyde; ICA, indole-3-carboxylic acid.

Indole metabolites have also been found to be used in adjuvant cancer therapy, especially immunotherapy led by immune checkpoint inhibitors. Immune checkpoint blockade (ICB) such as anti-programmed cell death protein 1 antibody (αPD-1), although it can restart the host’s T-cell immunity, is still poorly efficacious in the majority of patients in practice, especially in the portion of patients whose tumor genotypes are shown to be Microsatellite stabilized (MSS).^34^ Our recent study reveals that ILA produced by the intestinal commensal *Lactobacillus johnsonii* is further metabolized to IPA by *Clostridium sporogenes* in the gut^14^ (Figure 3A). IPA that enters the tumor microenvironment modulates immune cell stemness by modulating histone acetylation and enhances the efficacy of αPD-1 immunotherapy in melanoma, breast cancer, and colorectal cancer. Specifically, IPA enhances acetylation of histone 3 lysine 27 (H3K27ac) in the *Tcf7* super-enhancer region to up-regulate the proportions of TCF-1^+^ progenitor exhausted CD8^+^ T cells, which play a key role in the immunotherapy process.

Little is known about the exact role of interactions between microbial indole metabolites and host epigenetic mechanisms. As mentioned above for ILA and IPA, indole metabolites appear to have a broad regulatory effect on histone acetylation in immune cells. Whether indole metabolites act like short-chain fatty acids to modulate the enzymatic activity of the histone deacetylases family (HDACs) is not clear.^35,36^ Although ILA can enhance the level of H3K27ac at the IL12a enhancer regions to promote IL12a production in dendritic cells, this effect is not mediated by inhibiting the expression of HDACs.^17^ It also remains to be elucidated whether the histone acetyltransferases family (HATs) and acetyl coenzyme A are involved in indole metabolite-mediated regulation of histone acetylation. Whether IPA may function by acting as an inhibitor of HDACs or an agonist of HATs requires further investigation, such as enzyme activity assays. In the future, analysis of the mode and site of indole metabolites on key regulatory enzymes based on molecular docking and surface plasmon resonance (SPR) techniques may be a breakthrough that structural biologists can attempt.

#### 3.2.2. IAld: a microbial derived AhR ligand

IAld was found to be produced by *Lactobacillus reuteri* and translocated ectopically into melanoma.^37^ IAld activates AhR in CD8^+^ T cells and promotes the transcription of effectors such as IFNγ by phosphorylating CREB to sensitize αPD-L1 immunotherapy (Figure 3B). The authors also found in their clinical cohort that advanced melanoma patients who responded to immunotherapy had higher serum IAld levels. And patients with high IAld had longer progression-free survival (PFS) and overall survival.^37^ The application of ICB often brings some side effects to patients. As a common and serious immune-related adverse events (irAE), immune checkpoint inhibitor (ICI)-induced colitis causes inflammatory cell infiltration and extensive inflammation, even leading to termination of the patient’s ICB therapy.^38^ IAld could also attenuate ICI-induced colitis in mice by modulating the gut microbiota, host AhR/IL-22 axis and IL-10^+^ regulatory T (Treg) cells.^39^ It should be noted that IAld may be further metabolized to ICA or indole-3-carbinol in the host, and whether these potential effects are synergistic with these downstream metabolites deserves attention in future experiments.

#### 3.2.3. ICA: a partial agonist of AhR

In addition to producing ILA to inhibit CRC, *Lactobacillus gallinarum* was found to metabolize tryptophan to produce IAld, which was further metabolized to ICA.^40^ Kyn is known to promote Foxp3^+^ CD25^+^ Treg cells development. ICA could inhibit the expression of indoleamine 2,3-dioxygenase (*IDO1*) to reduce the release of Kyn and the infiltration of Treg cells in the tumor microenvironment. The authors also found that ICA can act as a partial agonist of AhR, antagonizing downstream Foxp3 in the presence of AhR agonists such as Kyn, further decreasing the abundance of Treg cells to improved αPD-1 efficacy (Figure 3C).

ICA is a good example of how not all indole metabolites are full agonists of the AhR. Partial agonists are dual agonists and antagonists, producing weak agonism when used alone or in combination with receptor antagonists. The authors found by SPR that ICA itself has a higher receptor affinity for AhR compared to Kyn, although with a weaker intrinsic activity.^40^ This mode of competition and cooperation for AhR binding by different indole metabolites also deserves further investigation.

#### 3.2.4. IAA: a neoadjuvant molecule for tumor chemotherapy

Chemotherapy is the most commonly used treatment for metastatic pancreatic ductal adenocarcinoma (PDAC),^41^ but PDAC has a response rate of less than 50% to first-line chemotherapy regimens.^42^ Although tumor markers such as CA19–9 have been shown to be useful in predicting the efficacy of response to chemotherapy, there is a lack of observables for long-term response.^43^ Correlations between the gut microbiota and the long-term survival of chemotherapy for PDAC have been revealed,^44^ but the exact components in the microbiota that affect the survival remain unclear.

In a recent study, IAA produced by *Bacteroides fragilis* and *Bacteroides thetaiotaomicron* was found to be highly abundant in the serum of PDAC-sensitized patients and was significantly correlated with overall survival.^45^ IAA can be metabolized by myeloperoxidase in neutrophils, and in combination with the FIRINOX (5-FU, irinotecan and oxaliplatin) induces the accumulation of reactive oxygen species and alteration of autophagic pathways in tumor cells to counteract PDAC (Figure 3D). But a recent study reported that IAA could disrupt mitochondrial function in intestinal stem cells, leading to impaired differentiation.^46^ The authors also found that fecal IAA was higher in people with mental illness and was associated with intestinal dysfunction. Further safety evaluations are needed for the future use of IAA in clinical patients.

## 4. Conclusion and future perspectives

In summary, a variety of indole metabolites have different effects on tumor development, which can modulate the tumor death mode or target immune cell subsets in the microenvironment, or can be adjuvant to tumor immunotherapy and chemotherapy (Table 1). We summarize some highlights of indole metabolites regulating the tumor microenvironment:

- The same indole metabolite can be metabolized from the same substrate catalyzed by different microbial enzymes.
- Indole metabolites can not only directly inhibit tumor growth, but also regulate multiple components in the immune microenvironment.
- The same indole metabolite may play completely opposite roles in different solid tumors.
- Indole metabolites can act as natural ligands for a variety of host receptors and have the potential to broadly regulate host epigenetic states.
- Indole metabolites can participate in the ferroptosis process of tumors and help respond to tumor immunotherapy and chemotherapy.

**Table 1.** The role of microbial indole metabolites in tumor.

Indole metabolites	Commensal	Tumor types targeted	Directly acting cells	Effects	Ref
ILA	*Lactobacillus gallinarum*	Colorectal cancer	Cancer cells	Directly inhibit cancer cell proliferation and promote apoptosis through AhR	15
ILA	*Lactobacillus plantarum*	Colorectal cancer	Dendritic cells/CD8^+^ T cells	Promote the production of IL-12 in dendritic cells and inhibit the *Saa3* in CD8^+^ T cells	17
ILA	*Lactobacillus reuteri*	Colorectal cancer	Th17 cells	Reduce Th17 cells by inhibiting the transcriptional activity of RORγt	18
ILA	*Bifidobacterium breve*	Colorectal cancer	Macrophages	Reduce the proportion of CD86^+^ pro-inflammatory macrophages through the AhR/p-AKT/IL-1b pathway	19
IPYA	*Prevotella copri*	Breast cancer, cervical cancer	Cancer cells	Directly inhibit the transcription of UHRF1 and suppress the growth of cancer cells in an AMPK signaling pathway-mediated manner	25
IDA	*Peptostreptococcus anaerobius*	Colorectal cancer	Cancer cells	Upregulate ALDH1A3 expression and help ferroptosis-suppressor protein 1 produce reduced coenzyme Q10	33
IPA	*Lactobacillus johnsonii+ Clostridium sporogenes*	Melanoma, breast cancer, colorectal cancer	CD8^+^ T cells	Enhance H3K27ac in the *Tcf7* super-enhancer region to up-regulate the proportions of TCF-1^+^ progenitor exhausted CD8^+^ T cells	14
IAld	*Lactobacillus reuteri*	Melanoma	CD8^+^ T cells	Promote the transcription of IFNγ by phosphorylating CREB in CD8^+^ T cells	37
ICA	*Lactobacillus gallinarum*	Colorectal cancer	Treg cells	Act as a partial agonist of AhR, antagonizing downstream *Foxp3* in the presence of Kyn	40
IAA	*Bacteroides fragilis, Bacteroides thetaiotaomicron*	Pancreatic ductal adenocarcinoma	Neutrophils	Be metabolized to toxic molecules by myeloperoxidase in neutrophils	45

However, outstanding questions remain to be addressed, such as the screening and identification of indole metabolites, their downstream mechanisms of action, and applying them to the clinic. The first question is how to identify and find new indole metabolites of microbial source. The currently known indole metabolites may be only a fraction of what microbes produce by metabolizing tryptophan. In different tumor types, the microbiota tends to produce different kinds of indole metabolites. It is also possible that primary and metastatic tumors contain different indole metabolites. Comprehensive multi-omics analysis and validation of metagenomics, spatial transcriptomics or metabolomics datasets from larger clinical cohort samples are required.

For clinical application and drug discovery, it may be good to have a blueprint of the spatial distribution of indole metabolites. Reference concentrations of indole metabolites in feces, blood, and organs of different human races or special populations need to be established. In addition, further pharmacokinetic studies are essential. Each indole metabolite has its own bioavailability. Their absorption, metabolism, and excretion characteristics, their local onset concentrations in different tumors, and the optimal route of administration need to be further confirmed. This global indole metabolite profile will lay the foundation for future clinical applications in the tumor prevention and treatment.

In terms of mechanism of action, the role of AhR deserves further attention. Indeed, many articles have reported that high expression of AhR in tumor cells seems to be associated with tumor progression and poor prognosis, whereas many indole metabolites often exert their tumor-suppressive effects through AhR.^27,47^ As mentioned above, the complex intrinsic mechanism may involve full and partial agonism of the receptor. The affinity and specificity of different indole metabolites for AhR need to be further elucidated. Notably, recent articles have also reported that indole metabolites can act on other receptors in a non-AhR-dependent manner. For example, IPA can repair gastrointestinal barrier function^48^ and liver damage^49^ via pregnane X receptor (PXR). Zhao X et al. reviews in detail the possible protein targets of bacterial-derived indole metabolites, in particular that aromatic monoamines can bind the orphan receptor GPRC5A.^50^ Another study found that knockout mice for the intestinal G-protein-coupled receptor 35, showed a decrease in IAld and an increase in ILA.^51^ Furthermore, in a mouse model of *Citrobacter rodentium* infection, indole-3-ethanol, IPYA and IAld can activate dopamine receptor D2 (DRD2) to produce colonization resistance and protect the host.^52^ The relationship between these receptors and the prognosis of tumors needs to be explored in further experiments.

For future research trends, the cooperation and competition between bacteria and indole metabolites need additional attention. Different bacteria could produce different indole metabolites, and it is worth considering how to precisely regulate the ratio of indole metabolites in the tumor microenvironment via diet^53^ or synthetic materials.^54,55^ Although the current role of indole metabolites on tumors is mostly focused on CRC, the role in other solid tumors such as hepatocellular carcinoma^56^ has not been revealed. Recent literature has also reported chemical ways to track tryptophan moieties, which opens up endless possibilities for manipulating the metabolic flow of tryptophan.^53,57^ In addition to bacteria, Fungi also contain tryptophan metabolizing enzymes.^58^ Like the dark matter in the microbiota, the specific indole metabolites produced by non-bacterial microbes and their effects on tumors deserve further attention. Finally, clinical trials using specific bacterial strains to intervene in tumor ICB immunotherapy have also shown promising results.^5,59^ The role of indole metabolites in other types of immunotherapies such as chimeric antigen receptor T-cell therapy (CART), and their impact on severe irAE will be a definite trend for future research. In conclusion, indole metabolites play an indispensable role in the tumor microenvironment, and it is foreseeable that in-depth investigation of the functions and mechanisms of indole metabolites will potentially bring new therapeutic values.

## Abbreviations

AhR: Aryl hydrocarbon receptor
ALDH1A3: aldehyde dehydrogenase 1 family member A3
αPD-1: anti-programmed cell death protein 1 antibody
ArAT: aromatic amino acid aminotransferase
CART: chimeric antigen receptor T-cell therapy
CRC: colorectal cancer
DRD2: dopamine receptor D2
fldH: phenyllactate dehydrogenase
FMT: fecal microbiota transplantation
H3K27ac: acetylation of the histone 3 lysine 27 site
HATs: histone acetyltransferases family
HDACs: histone deacetylases family
IA: Indole Acrylic Acid
IAA: Indole Acetic Acid
IAAld: Indole-3-Acetaldehyde
IAld: Indole-3-Aldehyde
IAM: Indole-3-Acetamide
ICA: Indole-3-Carboxylic Acid
ICB: Immune checkpoint blockade
ICI: Immune checkpoint inhibitor
IDA: *trans*-3-indoleacrylic acid
IDO1: indoleamine 2,3-dioxygenase
ILA: Indole-3-Lactic Acid
IL4I1: interleukin 4-induced 1
IPA: Indole-3-Propionic Acid
IPYA: Indole-3-Pyruvate
irAE: immune related adverse events
Kyn: kynurenine
ldhA: D-2-hydroxyacid dehydrogenase
MSS: Microsatellite stabilized
PDAC: Pancreatic ductal adenocarcinoma
PFS: progression-free survival
PXR: pregnane X receptor
SPR: surface plasmon resonance
Th17: T helper 17 cells
TMO: Tryptophan 2-Monooxygenasee
Treg: regulatory T cells
